# The impact of sex hormone concentrations on decision-making in females and males

**DOI:** 10.3389/fnins.2014.00352

**Published:** 2014-11-05

**Authors:** Birgit Derntl, Nina Pintzinger, Ilse Kryspin-Exner, Veronika Schöpf

**Affiliations:** ^1^Department of Psychiatry, Psychotherapy and Psychosomatics, RWTH Aachen UniversityAachen, Germany; ^2^Jülich Aachen Research Alliance (JARA BRAIN), Translational Brain MedicineJülich/Aachen, Germany; ^3^Institute for Neuroscience and Medicine (INM-1), Research Center JülichJülich, Germany; ^4^Faculty of Psychology, University of ViennaVienna, Austria; ^5^Department of Biomedical Imaging and Image-guided Therapy, Medical University of ViennaVienna, Austria

**Keywords:** sex, decision-making, testosterone, progesterone, estradiol, risk-taking

## Abstract

Human decision-making has been frequently studied and sex differences have been reported. Interestingly, previous results of hormone concentration on decision-making are somewhat inconsistent, regarding the impact of menstrual cycle phase in women or the influence of testosterone concentration on decision-making in women and men. However, the influence of the female sex hormone concentration (estradiol, progesterone) and the impact of oral contraceptive intake have rarely been examined and data regarding the effect of daytime variations of male testosterone are lacking. Moreover if personality factors such as sensation seeking, impulsivity, and anxiety influence decision-making, sex-specific effects, act as modulators is unclear. In the present study 71 women and 45 men were enrolled. All participants performed an evaluated decision-making task measuring risk-taking behavior on the basis of contingencies (Haegler et al., 2010), which can be carried out several times without a learning effect. Saliva samples were collected to obtain estradiol, progesterone, and testosterone levels. Additionally, all participants completed questionnaires measuring various personality factors. Data analysis revealed no sex differences in decision-making and no significant impact of testosterone concentration on behavioral performance in women or men. However, a significant negative correlation between progesterone concentration of women in the luteal phase and their performance in the risk-averse condition was obtained. Interestingly, a significant correlation between trait anxiety and decision-making occurred in females and males. Despite similar risky decision-making of women and men and no influence of testosterone concentration, menstrual cycle phase showed an effect on risk taking in women. In contrary to other studies, our findings provide rather subtle evidence for hormonal influences in decision-making, which may be primarily explained by task factors.

## Introduction

Every day is characterized by lots of decisions that we make, covering basic needs such as what to eat and drink and higher-order motives, e.g., who will I talk to during lunch break. In general, decision-making plays a pivotal role in our lives and comprises a complex process of assessing and weighing short-term and long-term costs and benefits of competing actions (van den Bos et al., 2012). The output of the decision-making process, i.e., which action is to be taken, is determined by an interaction between impulsive or emotionally based systems, responding to immediate (potential) rewards as well as losses or threats, and reflective or cognitive control systems controlling long-term perspective (Bechara, 2005).

One important factor of decision-making is risk taking, meaning the tendency of preferring an action with a possible large profitable or aversive outcome, although unlikely, over an alternative action with small profitable more likely outcome. Previous research in this regard has mostly demonstrated that women show less risk taking behavior than men in various domains (e.g., Jianakoplos and Bernasek, 1998; Byrnes et al., 1999; Zuckerman and Kuhlman, 2000; Zuckerman, 2006).

As pointed out by Stanton et al. (2011), economic risk is a domain that most individuals are frequently confronted with and thus of particular interest. One approach to measure an individual's propensity for risk taking in the face of monetary rewards and punishment is the Iowa Gambling Task (IGT, Bechara et al., 1994; Bechara, 2005). In this rather “economic” decision-making task participants learn to differentiate long-term advantageous from long-term disadvantageous decks of cards through exploration. Here, it is well-established that men and women differ in decision-making performance, with men choosing more cards from the long-term advantageous decks than women within the standard number of 100 trials (Bolla et al., 2004; Overman et al., 2006; Visser de et al., 2010). According to a recent review on sex differences in IGT performance by van den Bos et al. (2013), sex differences only emerge after about 60 trials, meaning that in the very beginning, females and males perform similar. Later on males seem to shift earlier to applying the correct rule by taking more cards from the long-term advantageous decks, while women need more time. In the end, both sexes prefer the long-term advantageous decks, however, women need longer before doing so consistently. As female reward sensitivity and processing are shaped by the menstrual cycle this could be related to the obtained sex difference, however, previous attempts to investigate this factor did not show a clear effect (Reavis and Overman, 2001; van den Bos et al., 2007).

Another crucial aspect might be testosterone concentration, which has been frequently linked particularly to risky decision-making. Recent data from Stanton et al. (2011) indicate that females and males with high testosterone levels show more risky behavior than those with low testosterone concentration, with a more pronounced effect in women. Besides age effects (e.g., Diver et al., 2003), it has been argued that testosterone concentration fluctuates across the day, with higher values after awakening than in the afternoon or evening in males (Axelsson et al., 2005). Until now it is unclear whether this diurnal variation influences decision-making and particularly risk taking.

Moreover, other studies in humans employing decision-making paradigms such as the Game of Dice Task (Starcke et al., 2008) and the Balloon Analog Risk Task (Lighthall et al., 2009, 2011) have not observed differences between men and women regarding risk-based decision-making. Furthermore, in the Cambridge Gambling Task men and women did not differ in risk-taking or impulsivity, but only in risk-adjustment, i.e., adjusting betting behavior according to the likelihood of winning (Deakin et al., 2004; van den Bos et al., 2014).

At present therefore it is not exactly clear under which task conditions men and women differ in decision-making and how this relates to differences in sex hormone concentration due to menstrual cycle phase (progesterone, estradiol) or daytime (testosterone). Such knowledge however will give more insight in how and under which circumstances sex differences in decision-making can be observed.

The aim of the present study therefore was to investigate the impact of (a) menstrual-cycle phase vs. oral contraceptive intake, (b) diurnal variation of testosterone in males and (c) testosterone concentration in females and males on decision-making. Besides group differences, we also analyzed potential associations between behavioral performance, hormonal parameters, and self-report questionnaire date.

## Methods and materials

### Sample

Seventy-one right-handed healthy females aged 19–37 years (mean age 23.8 years, *SD* = 3.7) participated in the study. When contacted, female participants were asked whether they were taking oral contraceptives and if not, were asked to report their menstrual cycle phase and cycle duration. Based on this information they were assigned a testing date. Only females who reported regular cycle duration (range: 25–35 days, mean days = 28.3, *SD* = 2.5) were included. At the day of testing, 22 females were in their follicular phase (days 1–12 of menstrual cycle; FO; mean age 23.6 years, *SD* = 3.8), 26 were in their mid-luteal phase (days 18–25 of menstrual cycle; LU, mean age 24.3 years, *SD* = 3.8) and 23 were taking oral contraceptives (OC, mean age 23.3 years, *SD* = 3.5). All females were tested between 9 and 11 a.m.

Moreover, 45 right-handed males aged 20–36 years (mean age 24.8 years, *SD* = 3.1) were tested. Twenty-two were tested before noon (9 to 11 a.m.) when testosterone levels are supposed to be higher (mean age 24.4 years, *SD* = 2.0), while the other 23 were measured in the late afternoon between 5 and 7 p.m. (mean age 25.1 years, *SD* = 3.8).

Groups did not differ in age [*F*_(4, 111)_ = 1.001, *p* = 0.410], or educational level [*F*_(4, 111)_ = 1.148, *p* = 0.338].

Additionally, all participants were asked to fill out several questionnaires tapping verbal intelligence (Mehrfachwahl-Wortschatz-Intelligenztest Version B, MWT-B, Lehrl, 2005), sensation seeking (SSS-V, Zuckerman, 1994), impulsivity (Barrett Impulsivity Scale, German Version: Preuss et al., 2008), depression (Beck Depression Inventory II BDI, Beck et al., 2006) and anxiety (State trait anxiety inventory, STAI, Laux et al., 1981).

Participants were recruited by advertisements at the University of Vienna and the Medical University of Vienna, Austria. All participants were screened for history of any psychiatric or mental disorder by using the German version of the structured interview of DSM IV (SCID; Wittchen et al., 1997). Written informed consent was obtained from all subjects prior to the examination and the study was approved by the local institutional review board.

### Saliva samples

To obtain actual estradiol, progesterone and testosterone levels saliva samples were collected on the day of testing. Saliva samples have been shown to have great potential for studying ovarian and androgen hormone levels as a reliable, feasible, and non-invasive method (e.g., Gandara et al., 2007). Before we started obtaining saliva samples we asked participants to wash out their mouth with water. In order to avoid arbitrary results we collected saliva samples for each hormone every half hour, thus we collected three samples per hormone in total (multiple sampling). Participants were instructed to fill a small plastic vial with at least 1.5 ml saliva (max. 3 ml) using a straw to stimulate saliva flow. Participants' collection vials were sealed after each collection and frozen immediately in accordance with previous research on sample storage (see Gröschl, 2008).

Saliva samples were analyzed by the European Institute for Salivary Analysis (Swiss Health Med, Aying, Germany) using an enzyme-linked immunoassay method from DRG (DRG Marburg, Germany; Salivary Estradiol ELISA SLV-4188, DRG Salivary Progesterone ELISA SLV-2931, DRG Salivary Testosterone ELISA SLV-3013). Analytical sensitivity (confidence interval 95%) was 0.4 pg/mL (Estradiol), 3.9 pg/mL (Progesterone), and 1.9 pg/mL (Testosterone). For estradiol, intra- and inter-assay coefficients were 3.8 and 2.6%, respectively. For Progesterone, intra- and interassay coefficients were 7.7 and 5.3%, respectively. For testosterone, intra-assay coefficients were <4% and inter-assay CV < 5%.

For details on hormone concentration of groups see Table 1.

**Table 1:** **Description of groups including sociodemographic, hormonal, and neuropsychological means (standard deviations in parentheses) and *p*-values**.

**Females**	**Follicular (*n* = 22)**	**Luteal (*n* = 26)**	**Oral contraceptives (*n* = 23)**	***p*-values**
Age	23.6 (3.8)	24.3 (3.8)	23.3 (3.5)	0.610
Estradiol (pg/mL)	3.9 (1.3)	5.6 (7.2)	3.9 (1.4)	0.351
Progesterone (pg/mL)	94.4 (113.1)	197.1 (133.3)	65.6 (21.7)	**<0.001**
MWT-B (raw score)	28.1 (3.4)	28.4 (3.2)	28.7 (3.0)	0.932
TMT-A (raw score)	19.5 (3.8)	20.2 (6.9)	20.1 (4.8)	0.886
TMT-B (raw score)	36.1 (10.0)	36.6 (13.0)	35.6 (10.1)	0.949
**Males**	**Morning (*n* = 22)**	**Afternoon (*n* = 23)**	***p*-values**	
Age	24.4 (2.0)	25.1 (3.8)	0.409	
Testosterone (pg/mL)	70.0 (20.7)	56.6 (17.6)	**0.023**	
MWT-B (raw score)	27.3 (2.5)	28.8 (2.4)	**0.047**	
TMT-A (sec)	18.6 (6.6)	19.9 (5.9)	0.483	
TMT-B (sec)	36.0 (13.5)	34.7 (9.3)	0.710	
**Testosterone**	**Females HT (*n* = 36)**	**Females LT (*n* = 35)**	**Males HT (*n* = 22)**	**Males LT (*n* = 23)**
Age	23.7 (4.0)	23.8 (3.3)	24.3 (2.2)	25.2 (3.7)
Testosterone (pg/mL)	12.9 (11.7)	2.7 (1.5)	79.9 (12.8)	47.2 (10.5)

Significant p-values are marked in bold.

Note: MWT-B, Mehrfachwortwahltest-B measures verbal intelligence; TMT-A/-B, Trail Making Test -A/-B measure executive functions. HT, high testosterone concentration; LT, low testosterone concentration.

### Decision-making task

For this study we chose out of a battery of decision-making tasks which can be performed repeatedly without learning effect. Such tasks include for example the Balloon Analog Risk Task (Lejuez et al., 2002) the Cambridge cognition task (http://www.cambridgecognition.com/), the Game of Dice Task (Brand et al., 2005), and the Haegler's Risk Game (HRG). The HRG is based on a card game which is described in great detail elsewhere (Haegler et al., 2010). Briefly, participants were told that they would see an unknown amount of play card pairs with values from 1 to 10, 1 being the smallest and 10 being the highest possible card. After seeing the first card, participants had to decide whether the second card, would be either higher or lower than the first card. If their choice was correct, participants gained reward points. If their choice was wrong, participants lost points.

Starting with 0 points, reward points were accumulated over the rounds, while it was also possible to accumulate a negative amount of points. Participants were instructed that reward points were valuable, and it was the goal of the game to accumulate as many points as possible. They were paid a fixed amount of money, which they were aware of before the study started, but there was no mapping between points and monetary reward. Nevertheless, participants were instructed to play the HRG with the objective of winning as many points as they could. In total, 100 card pairs were presented per game cycle, taking approximately 5 min for completion. The first card was pseudo-randomized and ranged from 2 to 9. The second card was selected by chance ranging from 1 to 10 but always occupying a different value than the first card. Presentation of the first card was accompanied by additional information: the amount of points to be won if the participants' choice was correct was shown in green ink; the amount of points that could be lost was shown in red ink. Additionally, a green–red bar indicated the ratio between the possible number of points to be won or lost. Participants indicated their choice by either pressing the lower or the higher button in the response panel. After making their choice the points were either added or subtracted from the total amount of points depending on the accuracy of the response. Additionally, the second card appeared highlighted by a green or red box in combination with a dialog window saying either “You win!” or “You loose!” depending on the accuracy.

Since the second card was drawn completely random, the statistical probability for the second card to be lower varied according to the value of the first card. As an example, if the first card carried the value 2, the probability for the second card to be lower was 1/9. If the first card carried the value 9, the probability for the second card to be lower was 8/9. The amount of points to be won or lost for a correct or incorrect choice varied and was directly correlated to the statistical likelihood of the event to occur. The probability of the second card to be higher if the first card carried a value *x* ∈ 2, …, 9 was *p*_higher_ = (10 − x)/9, therefore, the points that could be lost were (10 − *x*) × 10 and the points that could be gained were 90 − [(10 − *x*) × 10]. For the second card to be lower, the probability was *p*_lower_ = 1 − *p*_higher_, resulting in either a deficit of 90 − [(10 − *x*) × 10] points or a debit of (10 − *x*) × 10 points.

Due to the fact that the points to be won or lost were opposed to the probabilities, the chances of winning or loosing were random, resulting on average in a total amount of 0 points at the end of the game cycle. Hence, no strategy could be learned which would help the participants to win the game. Thus, in contrast to other gambling games like for instance the IGT, participants can play the HRG multiple times without a learning effect.

**Figure 1:**
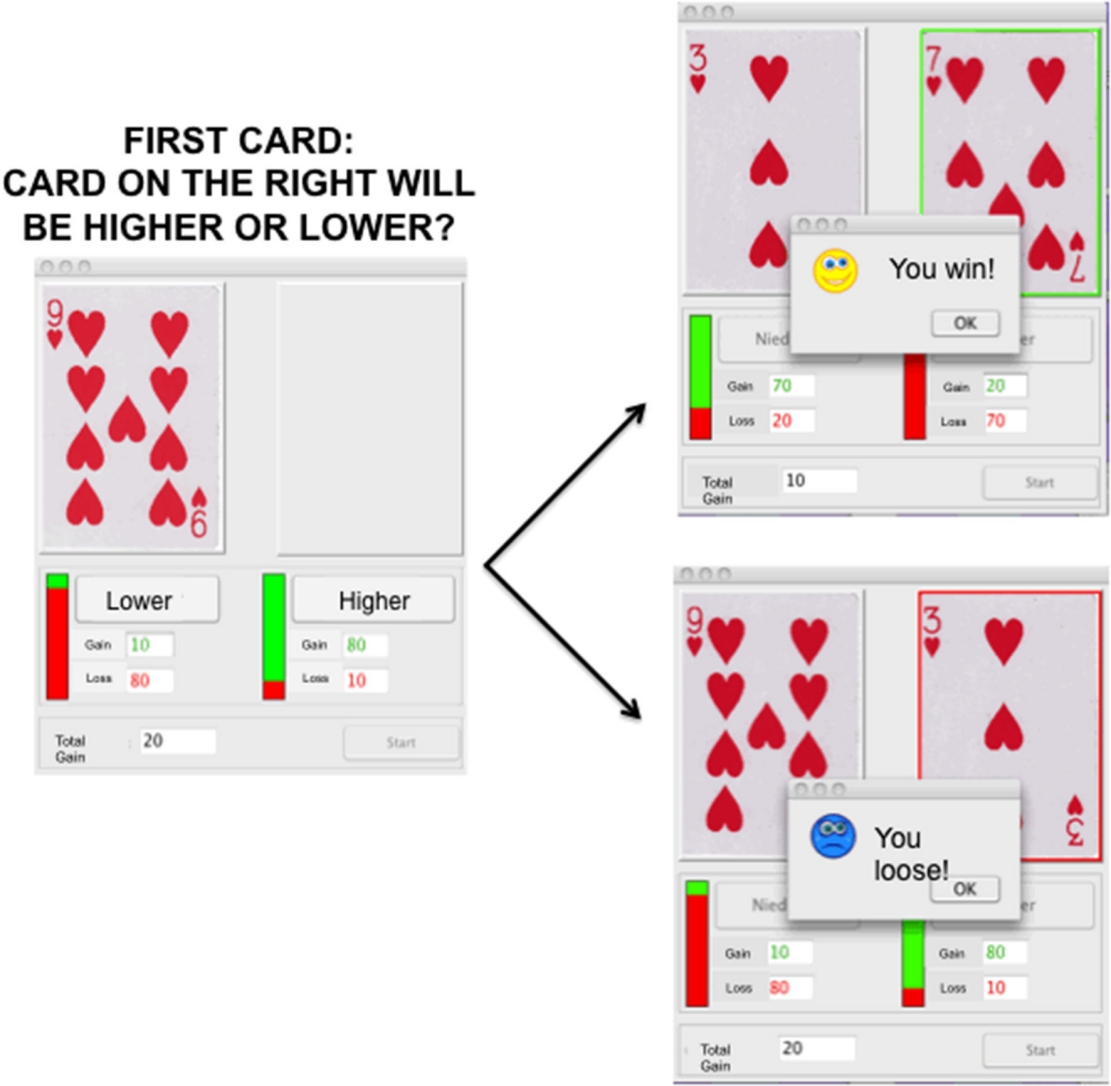
**Illustration of Haeglers Risk Game depicting the screen with one card on the left and the option of the participant to choose a lower or higher card will be displayed on the left**. On the right the two alternatives, depicting either a win (top), or a loose (bottom) trial, are illustrated.

Participants were considered as playing more risky if they chose higher while the first card was 6, 7, 8, or 9 or if they chose lower while the first card was 2, 3, 4, or 5 more often. The key dependent variable was, therefore, the summed number of risky selections of each participant. Accordingly, the pairs 2-lower and 9-higher, 3-lower and 8-higher, 4-lower, and 7-higher, as well as 5-lower and 6-higher were combined by summing up the number of single selections, due to equal probabilities. This resulted in a total of 4 risk values per participant. On average each card value of the first card appeared 12.5 times during a game cycle, hence, the average number of presentations of one card pair was 25 per game cycle. During each game cycle the response time, meaning the time from the display of the first card until participants pressed either the higher or the lower button, as well as each choice made by the participants were monitored. Please see Figure 1 for illustration of the task.

### Statistical analysis

Statistical analyses were performed using SPSS 20.0 and level of significance was set at *p* = 0.05. We performed three different analyses in order to compare the three female groups (FO vs. LU vs. OC), the two male groups (morning vs. afternoon) and—in line with the paper by Stanton et al. (2011)—the impact of testosterone level (high vs. low concentration in females and males).

Number of card selections and reaction times in the HRG were analyzed using mixed-model ANOVAs with risk selection as within-subject factor and group as between-subject factor. For significant effects partial-eta squares are listed as estimates of effect size. In cases of violations of sphericity, statistical tests involving the risk selection factor employed Greenhouse-Geisser correction. All *post-hoc* results were Bonferroni corrected.

Group differences regarding neuropsychological parameters (MWT-B, TMT) and the questionnaire data (BDI, STAI, SSS-V, BIS) were assessed using multivariate ANOVAs.

Correlations between behavioral performance [frequencies and reaction times of high risk (2_9) and low risk (5_6) selections], hormone concentration and self-report questionnaire measures (SSS, BIS, BDI, STAI) were computed testing two-sided for negative, respective positive correlations.

Since progesterone (FO: *p* = 0.007, LU: *p* = 0.326; OC: *p* = 0.893) and estradiol (FO: *p* = 0.811; LU: *p* = 0.002; OC: *p* = 0.469) levels were not normally distributed in the three female groups, we transformed the values taking the square root, which is an adequate tool to apply to right skewed data (Bortz, 1999). The transformed hormone values then were normally distributed (progesterone: FO: *p* = 0.072, LU: *p* = 0.343; OC: *p* = 0.917; estradiol: FO: *p* = 0.589; LU: *p* = 0.063; OC: *p* = 0.747) and thus were entered in further analyses. In the male group, testosterone concentration was normally distributed (morning: *p* = 0.879, afternoon: *p* = 0.737).

Following the study by Stanton et al. (2011), we distributed our female and male group into high and low testosterone groups via median split of testosterone concentration.

Pearson correlations were calculated to investigate the influence of sex hormone levels on the behavioral performance. To adjust for significant inter-hormonal correlations additional partial correlations were calculated, controlling for estradiol/progesterone influence on the correlation between performance and hormone levels, respectively. Moreover, estradiol:progesterone ratio was calculated and entered in the correlation analyses.

## Results

**Figure 2:**
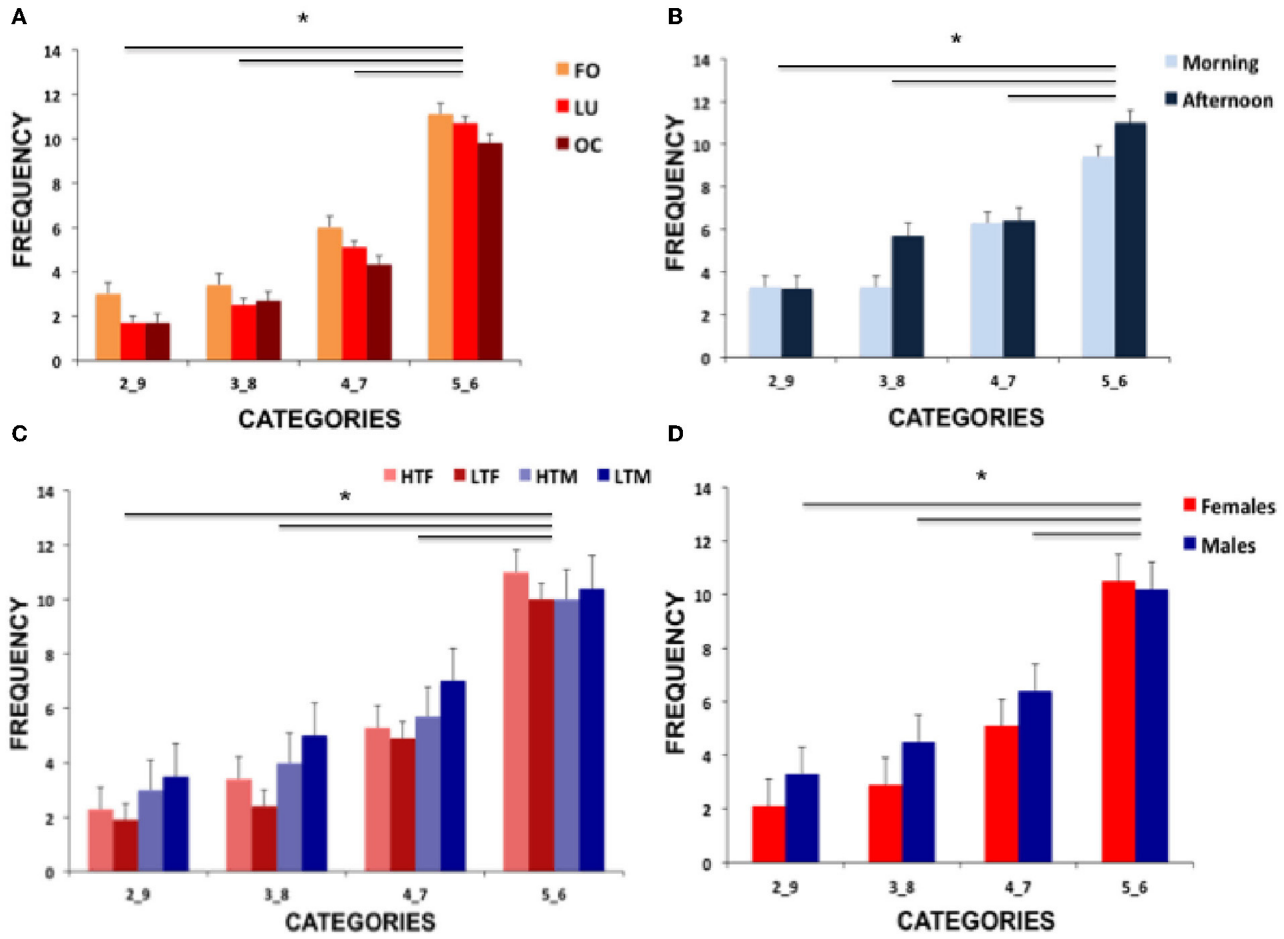
**Illustration of the results showing (A) frequencies of risk conditions for the three female groups (FO, follicular; LU, luteal; OC, oral contraceptive intake), (B) frequencies of the two male groups (morning and afternoon testosterone) and (C) frequencies of the high vs. low testosterone concentration females and males (HTF, high testosterone females; LTF, low testosterone females; HTM, high testosterone males; LTM, low testosterone males) and (D) comparison of performance of females and males**. Significant differences are marked with an asterisk.

Figure 2 displays performance of all group comparisons and Table 1 shows means and standard deviations of hormone concentration and neuropsychological parameters.

### Females

#### Hormone concentration

Females in the three groups showed significantly different progesterone levels [*F*_(2, 68)_ = 12.700, *p* < 0.001, part-eta sq. = 0.272]. *Post-hoc* analysis showed that LU females had significantly higher progesterone levels than both other groups (LU vs. FO: *p* = 0.001; LU vs. OC: *p* < 0.001). No group difference emerged for estradiol [*F*_(2, 68)_ = 1.145, *p* = 0.324]. Table 1 (top section) shows means and standard deviations of hormone concentration.

#### Decision-making

Applying a mixed-model ANOVA with risk selection as within-subject factor and group (FO vs. LU vs. OC) as between-subject factor, we observed a significant risk selection effect [*F*_(1.741, 118.393)_ = 134.049, *p* < 0.001, part-eta-sq. = 0.663], no significant group effect [*F*_(1, 68)_ = 0.668, *p* = 0.516] and no significant interaction [*F*_(1.741, 118.393)_ = 0.321, *p* = 0.839].

*Post-hoc* tests disentangling the significant risk selection effect revealed that the highest number of selections was present for the least risky parameters (5_6) and the lowest number of selections was present for the most risky parameters (2_9, all *p-values < 0.019*). See Figure 2A for illustration of results.

Regarding reaction times, we focused on the high (2_9) and low (5_6) risk conditions. The mixed-model ANOVA revealed no significant effect of risk selection [*F*_(1, 71)_ = 0.214, *p* = 0.647], no significant group effect [*F*_(1, 71)_ = 0.874, *p* = 0.428], and no significant risk-by-group interaction [*F*_(1, 71)_ = 0.950, *p* = 0.398].

#### Neuropsychological performance and questionnaire data

Females in the three groups did not differ in neuropsychological parameters (MWT-B: *p* = 0.647; TMT-A: *p* = 0.769; TMB-B: *p* = 0.922), or in the questionnaire data (all *p*-values >0.195). See also Table 1 (top section) for detailed information.

#### Correlation analyses between behavioral performance and hormone concentration

Analysis of a significant association between behavioral performance (risk selection and reaction times) and hormone concentrations (estradiol, progesterone) revealed a significant correlation between progesterone and reaction time in the high risk condition in LU (*r* = 0.471, *p* = 0.048), indicating faster responses in females with lower progesterone concentration. No other significant correlations emerged. For details see Table 2.

**Table 2:** **Overview on correlations between hormone concentrations and behavioral performance (selection as well as reaction times) for all groups**.

	**Selection**		**Reaction time**	
	**HGR 2_9**	**HGR 5_6**	**HGR 2_9**	**HGR 5_6**
**Follicular**				
*Estradiol*	*r* = −0.150, *p* = 0.505	*r* = 0.018, *p* = 0.936	*r* = 0.054, *p* = 0.855	*r* = 0.312, *p* = 0.299
*Progesterone*	*r* = −0.154, *p* = 0.493	*r* = −0.289, *p* = 0.192	*r* = −0.127, *p* = 0.666	*r* = −0.133, *p* = 0.665
*E:P*	*r* = 0.007, *p* = 0.976	*r* = 0.359, *p* = 0.101	*r* = 0.292, *p* = 0.312	*r* = 0.340, *p* = 0.256
**Luteal**				
*Estradiol*	*r* = −0.123, *p* = 0.550	*r* = 0.029, *p* = 0.888	*r* = −0.189, *p* = 0.626	*r* = −0.255, *p* = 0.507
*Progesterone*	*r* = −0.135, *p* = 0.510	*r* = −0.315, *p* = 0.117	***r* = 0.471, *p* = 0.048**	*r* = 0.442, *p* = 0.234
*E:P*	*r* = 0.052, *p* = 0.802	***r* = 0.657, *p* = 0.019**	***r* =−.530, *p* = 0.026**	*r* = 0.400, *p* = 0.257
**Oral contraceptives**				
*Estradiol*	*r* = 0.103, *p* = 0.638	*r* = 0.316, *p* = 0.141	*r* = 0.113, *p* = 0.755	*r* = −0.282, *p* = 0.429
*Progesterone*	*r* = −0.125, *p* = 0.569	*r* = 0.072, *p* = 0.744	*r* = 0.111, *p* = 0.760	*r* = −0.337, *p* = 0.341
*E:P*	*r* = 0.223, *p* = 0.305	*r* = 0.267, *p* = 0.217	*r* = 0.037, *p* = 0.919	*r* = 0.109, *p* = 0.765
**Morning group M**				
*Testosterone*	*r* = 0.163, *p* = 0.468	*r* = −0.053, *p* = 0.814	*r* = 0.509, *p* = 0.075	*r* = 0.226, *p* = 0.457
**Afternoon group M**				
*Testosterone*	*r* = −0.281, *p* = 0.194	*r* = 0.025, *p* = 0.910	*r* = −0.113, *p* = 0.688	*r* = −0.263, *p* = 0.344
**High testosterone F**				
*Testosterone*	*r* = −0.028, *p* = 0.871	*r* = 0.072, *p* = 0.678	*r* = −0.097, *p* = 0.711	*r* = −0.298, *p* = 0.263
**Low testosterone F**				
*Testosterone*	*r* = 0.013, *p* = 0.942	*r* = 0.188, *p* = 0.280	*r* = 0.040, *p* = 0.882	*r* = 0.037, *p* = 0.891
**High testosterone M**				
*Testosterone*	*r* = 0.138, *p* = 0.541	*r* = −0.170, *p* = 0.450	*r* = 0.065, *p* = 0.834	*r* = −0.201, *p* = 0.511
**Low testosterone M**				
*Testosterone*	*r* = −0.097, *p* = 0.661	*r* = 0.051, *p* = 0.818	*r* = −0.283, *p* = 0.307	*r* = −0.332, *p* = 0.227

Significant correlations are marked in bold.

Applying partial correlations did not reveal a significant correlation (all *p*-values > 0.054).

Relying on the estradiol:progesterone ratio revealed a significant correlation with low risk selection (HRG 5_6: *r* = 0.657, *p* = 0.019) as well as with reaction time in the high risk condition (HRG 2_9: *r* = −0.530, *p* = 0.026) again in LU. In FO and OC no correlation reached significance (all *p*-values > 0.107). For details please see Table 2.

#### Correlation analyses between behavioral performance and questionnaire data

In FO, trait anxiety correlated with high risk reaction time (*r* = 0.577, *p* = 0.016). In LU, the thrill and adventure score from the SSS-V (*r* = 0.808, *p* = 0.004) was significantly positively correlated with reaction time for the low risk selection. No other significant correlations emerged (all *p*-values > 0.051).

### Males

#### Hormone concentration

Testosterone levels were significantly different (*t* = 2.355, *p* = 0.023), with higher concentration in the morning group. Table 1 (middle section) shows means and standard deviations of hormone concentration.

#### Decision making

Applying a mixed-model ANOVA with risk selection as within-subject factor and daytime as between-subjects factor revealed a significant effect of risk selection [*F*_(1.849, 79.515)_ = 29.568, *p* < 0.001, part-eta-sq. = 0.407], no significant group effect [*F*_(1, 43)_ = 0.623, *p* = 0.434] and no significant interaction [*F*_(1.849, 79.515)_ = 1.145, *p* = 0.320]. *Post-hoc* analysis of the significant risk effect showed that high-risk selections were taken less often than the lower risk options (all *p*-values < 0.022).

Regarding reaction times of high and low risk selection, mixed-model ANOVA revealed no main effect of risk selection (*F*_(1, 26)_ = 0.271, *p* = 0.607], no main effect of group [*F*_(1, 26)_ = 1.670, *p* = 0.208], nor any interaction [*F*_(1, 26)_ = 1.798, *p* = 0.192]. See Figure 2B for results.

#### Neuropsychological performance and questionnaire data

Males did not differ in executive functioning (TMT-A: *p* = 0.483; TMT-B: *p* = 0.710) but in verbal intelligence (MWT-B: *p* = 0.047) with higher scores in the afternoon group. For details please see also Table 1 (middle section). Re-running the repeated-measures ANOVA with MWT-B as covariate did not change the results in risk selection or reaction times.

Regarding questionnaire data, males in the morning group reported higher trait anxiety (STAI-T, *p* = 0.037) than the afternoon group. All other comparisons remained not significant (all *p*-values > 0.376). Including STAI-T scores as covariate in the repeated-measures ANOVA did not influence significance and direction of the reported effects.

#### Correlation analyses between behavioral performance and hormone concentration

Analysis of a significant association between behavioral performance (risk selection and reaction times) and testosterone concentration revealed no significant associations (all *p-values >0.075*). For details see Table 2.

#### Correlation analyses between behavioral performance and questionnaire data

While scores in sensation seeking, impulsivity, and depression were not correlated with behavioral performance, trait anxiety scores were correlated with high-risk selection (*r* = 0.581, *p* = 0.002) in the morning group. Correlations with state anxiety did not reach significance (all *p*-values > 0.052) and no significant correlation emerged for the afternoon group (all *p-values > 0.068*).

### Females vs. males

#### Hormone concentration

Applying the median split, 23 males were divided in the low testosterone group (HTM, 9 from the morning group, 14 afternoon group), 22 males in the high testosterone group (LTM, 13 from the morning group, 9 afternoon group), 35 females in the low testosterone group (LTF) and 36 in the high testosterone group (HTF). LTF vs. HTF and LTM vs. HTM differed significantly in their testosterone levels (both *p*-values < 0.001). Table 1 (bottom section) shows means and standard deviations of testosterone concentration.

#### Decision making

Applying a mixed-model ANOVA with risk selection as within-subject factor and testosterone concentration as well as sex as between-subjects factor revealed a significant risk selection effect [*F*_(1.945, 217.857)_ = 127.116, *p* < 0.001, part-eta sq. = 0.532] but neither a testosterone effect [*F*_(1, 112)_ = 0.001, *p* = 0.970], nor a significant sex effect [*F*_(1, 112)_ = 1.506, *p* = 0.222] or interaction (all *p*-values > 0.142) occurred.

Regarding reaction times, no main effect of risk selection [*F*_(1, 56)_ = 0.065, *p* = 0.800] but a significant testosterone concentration effect [*F*_(1, 56)_ = 4.039, *p* = 0.049, part-eta sq. = 0.067] with faster responses in participants with low concentration and a trend for a sex difference [*F*_(1, 56)_ = 3.899, *p* = 0.053, part-eta sq. = 0.065] with faster reactions in females emerged. All interactions did not reach significance (all *p*-values > 0.298). See Figure 2C for results on testosterone groups and Figure 2D for results of females and males.

#### Neuropsychological performance and questionnaire data

Applying a multivariate ANOVA with testosterone concentration and sex as grouping factors revealed no significant main effect or interaction for verbal intelligence (MWT-B, all *p*-values > 0.256) or executive functioning (TMT-A, all *p*-values > 0.216; TMT-B, all *p*-values > 0.222).

Regarding questionnaire data, multivariate ANOVA again with sex and testosterone concentration as grouping factors demonstrated sex differences for the boredom susceptibility score [*F*_(1, 56)_ = 8.945, *p* = 0.004, part-eta sq. = 0.085], the thrill and adventure seeking score [*F*_(1, 56)_ = 6.432, *p* = 0.013, part-eta sq. = 0.063] as well as the total score of the sensation seeking scale [*F*_(1, 56)_ = 9.389, *p* = 0.003, part-eta sq. = 0.091] always with higher scores in males. Additionally for trait anxiety, females showed significantly higher scores than males (STAI-T, *F*_(1, 56)_ = 8.421, *p* = 0.005, part-eta sq. = 0.079]. For testosterone concentration no significant main effect (*p* = 0.060) or interaction with sex (*p* = 0.103) occurred and no other effect reached significance (all *p*-values > 0.060).

#### Correlation analyses between behavioral performance and hormone concentration

Analysis of a significant association between behavioral performance (risk selection and reaction times) and testosterone concentration in the separate groups (HTF, LTF, HTM, LTM) revealed no significant association (all *p*-values > 0.219). For details please see Table 2.

## Discussion

The present study aimed at analyzing the impact of menstrual cycle phase, diurnal testosterone variation, and testosterone concentration on decision-making relying on an evaluated task without learning effect, HRG (Haegler et al., 2010). Additionally, we investigated whether decision-making was associated with hormone concentration or personality and mood factors such as sensation seeking, impulsivity, depression or anxiety. This was realized by dividing the study cohort into three groups of females (follicular, luteal, and pill-taking) testing for cycle effects. The effect of diurnal variation of male hormone concentration was studied in two male groups (morning and afternoon measurement). Following the approach by Stanton et al. (2011), we investigated the impact of testosterone concentration on performance parameters in females and males. Notably, all participants were students thus groups had similar age and educational background. Moreover, they did not differ in basic neuropsychological parameters including verbal intelligence and executive functions.

The following section will be divided into different parts discussing menstrual cycle effects, influence of diurnal variation of testosterone on decision-making in males and the impact of high vs. low testosterone concentration in females and males. Moreover, a more general discussion and limitations of the conducted study will be reported.

### Menstrual cycle and decision-making

Previous studies reported heterogeneous findings regarding the impact of menstrual cycle phase and hormone concentration on decision-making: studies relying on self-report data frequently reported a significant rise in risk-taking behavior when estradiol levels were high (Chavanne and Gallup, 1998; Bröder and Hohmann, 2003; Haselton and Gangestad, 2006; Pillsworth and Haselton, 2006; Sukolová and Sarmány-Schuller, 2011), Saunders and Hawton (2006) reviewed several studies on suicide attempts and suicidal behavior in women and observed that during phases of low estradiol levels non-fatal suicidal behavior is more frequent, while for example Reavis and Overman (2001) or van den Bos et al. (2007) did not report a significant impact of menstrual cycle phase on performance using the Iowa Gambling task. Here, we also observed no significant difference in risk selection or reaction time between follicular and luteal females. Moreover, in contrast to previous studies we included pill-taking females but no significant group effect emerged. Further analyses of impact of hormone concentration revealed two significant findings in LU: while progesterone concentration was negatively correlated with reaction time for high risk selection, estradiol:progesterone ratio was positively associated with low risk selection. Hence, while we see no general impact of menstrual cycle phase, correlations with hormone levels, and behavioral performance occurred only in LU, where particularly progesterone levels were higher.

Our findings indicate that during the luteal phase, females showed faster responses for high risk options when their progesterone levels were higher but chose low risk options more often the higher their estradiol:progesterone ratio.

Our findings thus point out two different aspects: (1) using computerized experimental paradigms to assess risk selection revealed no significant impact of menstrual cycle, while studies relying on self-report data do. Therefore, one has to question whether the constructs assessed with one and the other might be different, have distinct values and relevance for the participants and whether a bias between self-report and experimental behavior exists. (2) analysis of hormone concentration showed some associations with behavioral performance, particularly with progesterone, supporting previous findings of more risk taking behavior in the luteal phase with higher progesterone values.

Evidence has accumulated that progesterone and its metabolites (mainly allopregnanolone, 3α,5α-THP) are important neuroactive steroids, which influence social, cognitive, and physical performance (for review see Frye, 2009; Pluchino et al., 2013). During the luteal phase, circulating concentrations of pregnanolone and 3α,5α-THP are 2–4 times higher than during the follicular phase (Purdy et al., 1990; Wang et al., 1996; Genazzani et al., 1998; Sundström and Bäckström, 1998a,b), with highest concentrations in the hippocampus and midbrain regions (Bixo et al., 1997). Typically, decision-making tasks elicit activation of prefrontal regions but also hippocampus activation has been reported (Li et al., 2010, for review see van den Bos et al., 2013). However, up to now the impact of concentration of progesterone and its metabolites on behavioral performance and neural activation underlying decision-making is still unclear. Hence, pregnanolone and 3α,5α-THP might also influence cycle-mediated performance in these tasks thereby contributing to the findings observed in previous studies as well as ours.

Additionally, we observed a significant positive correlation between trait anxiety and reaction time in the high risk condition only in the follicular group, indicating longer reaction times with higher trait anxiety. Hence, females during the follicular phase took longer to decide for the high risk option. This fits nicely with that assumption that high trait anxiety is linked with risk-avoidant decision making, which has been shown before (Broman-Fulks et al., 2014; Pittig et al., 2014). However, for sensation seeking and impulsivity, we only observed sparse associations with decision-making behavior. Several studies linked risk taking behavior with these personality factors (e.g., Mishra and Lalumière, 2011; Popham et al., 2011), however, others also failed to observe these associations (Bayard et al., 2011). Again, one possible factor explaining this divergence is the methodological variety in how decision-making or risk-taking was assessed. To further investigate these associations, future experiments might want to combine several approaches in order to highlight divergences and communalities.

Regarding oral contraceptive intake we failed to report any significant effect or correlation of hormone concentration with behavioral performance. Several factors might have influenced our findings, such as the heterogeneity of oral contraceptives taken by our women or the lack of information on duration of intake. This should be further investigated in future studies.

### Diurnal variation in testosterone and its impact on decision-making

Several studies linked testosterone concentration with risk-taking in that higher testosterone levels were associated with more risky behavior (e.g., Carney and Mason, 2010; Goudriaan et al., 2010). Despite the fact that we observed higher testosterone concentration in males measured in the morning compared to the afternoon, analysis of behavioral performance did not reveal a significant group effect and thus impact of the diurnal variation in testosterone on decision-making. Moffat and Hampson (1996) showed a significant difference in spatial processing between males tested at 8:15 vs. 10.15 a.m., with better performance in those with higher testosterone levels.

Interestingly, we observed a significant positive correlation of trait anxiety and high risk selection in the morning group, suggesting more risky decision making in males with higher trait anxiety. This finding contradicts a bulk of literature proclaiming less risk taking in high trait anxious individuals (Pittig et al., 2014). However, the three-way interaction of testosterone concentration, trait anxiety and decision-making performance in males has rarely been investigated, thus replications are necessary before conclusion can be drawn.

Additionally, in follicular females we observed a contradictory correlation, namely that higher levels of trait anxiety were associated with longer reaction times for risky selections, thus rather risk-aversive behavior. Sex differences in trait anxiety have been reported quite frequently, with higher values in females than males (Spielberger et al., 1983; McCleary and Zucker, 1991; Perkins et al., 2007). Moreover, sex-specific effects of trait anxiety on decision-making have also been reported before, suggesting sex-specific endophenotypes of anxiety which in turn affect cognitive functioning differentially (Visser de et al., 2010).

It remains an open question, which abilities are affected by diurnal variation and what role for instance seasonal variation of salivary testosterone concentration as shown by Stanton et al. (2011) plays regarding decision-making or more specifically, risk-taking.

### Testosterone concentration in females and males

Dividing females and males in groups with high and low testosterone concentration only revealed a significant testosterone effect for reaction times, with faster reactions in participants with lower testosterone concentration. As low risk options were selected most frequently by all participants this might partly support findings linking testosterone concentration and risk behavior. Interestingly, Stanton et al. (2011) showed that high-testosterone women and high-testosterone men made riskier choices than their low-testosterone counterparts of the same sex, and this effect was pronounced in women. Hence, the authors conclude that according to their findings high levels of testosterone are associated with willingness to incur greater risk in both sexes when using the IGT. In their review paper on sex differences in decision-making with a particular focus on studies using the IGT, van den Bos et al. (2013) resume that factors such as self-report vs. experimental modulation of risk taking behavior, acting in a group or acting alone or simply the fact that several studies investigating decision-making induced stress in females and males may lead to more risk-taking behavior in men. Notably, the authors conclude that previous data rather indicate no sex difference in immediate responses to emotional events, but only in the way these responses are regulated by for instance neuronal structures related to cognitive control.

### General discussion

Sex differences in the propensity to take risks have been documented in a large number of questionnaire and experimental studies (e.g., van den Bos et al., 2012). In a meta-analysis by Byrnes, Miller, and Schafer (1999) who reviewed over 150 papers on sex differences in risk taking, authors concluded that males are more likely to take risks than females. Notably, Figner and Weber (2011) pointed out that these sex differences in risk taking are domain-specific and can be explained by risk perceptions, which in turn are influenced by familiarity (Weber et al., 2005). Interestingly, once these differences in risk perceptions are taken into account, most of the sex differences in risk taking diminish as pointed out by Figner and Weber (2011). Here, we did not see a significant sex difference in risk taking as measured with the Haegler-Risk-Game (HRG). Following a domain-specific approach, it is hard to place the HRG, as there was no financial risk, no ethical risk, no recreational risk, no risk regarding health, or safety and no social decision risk, just the gambling risk with no economic consequences. Thus, we assume that risk perception was very low in females and males probably contributing to the lack of a general sex difference.

A potential influencing factor of the existing decision-making tasks is that they cannot be executed repeatedly without excluding a learning effect. Therefore, in the current study we relied on a novel computerized decision-making task in which participants had to make decisions between contingencies (Haegler et al., 2010). Due to the lack of winning strategy, the HRG can be played repeatedly without a learning effect. As learning behavior is modulated by hormone concentration particularly in the luteal phase (Andreano and Cahill, 2009), this might partly explain why we did not obtain significant group differences, instead only correlations with hormone concentration in the luteal phase.

Additionally, the study context also influences risky decision-making, with less consistent findings in laboratory settings (as in the study) as in field experiments (Eckel and Grossman, 2008). Although risky decision-making might be less consistent due to a laboratory setting, it has been discussed quite openly, that contextual conditions may introduce additional heterogeneity due to a gender interaction effect (Krajnik et al., 2014). Even in an animal model the effect of the experimenters sex on the baseline response in an androstadienone experiment, which is supposed to act as a chemosignal in humans, has recently been observed (Sorge et al., 2014). Also, another study in humans investigating the same compound, reported that the setting, the manner, and by whom the experiment was conducted played a role in perception (Lundström and Olsson, 2005). Especially for sex hormones it cannot be completely exluded that the experimenter collecting the samples might have an impact.

### Conflict of interest statement

The authors declare that the research was conducted in the absence of any commercial or financial relationships that could be construed as a potential conflict of interest.
